# Supplementary material for: Analysis of Long Non-Coding RNA in Cryptosporidium parvum Reveals Significant Stage-Specific Antisense Transcription
Source: Front Cell Infect Microbiol. 2021 Jan 14;10:608298. doi: 10.3389/fcimb.2020.608298 (PMC7840661; doi:10.3389/fcimb.2020.608298)
Supplement: Supplementary file 2 [file DataSheet_2.docx]

Supplementary Material

**Analysis of Long Non-coding RNA in *Cryptosporidium parvum* Reveals Significant Stage-Specific Antisense Transcription**

**Yiran Li^1^, Rodrigo P. Baptista^1,2^, Adam Sateriale^3#^, Boris Striepen^3^ and Jessica C. Kissinger^1,2,4,*^**

| 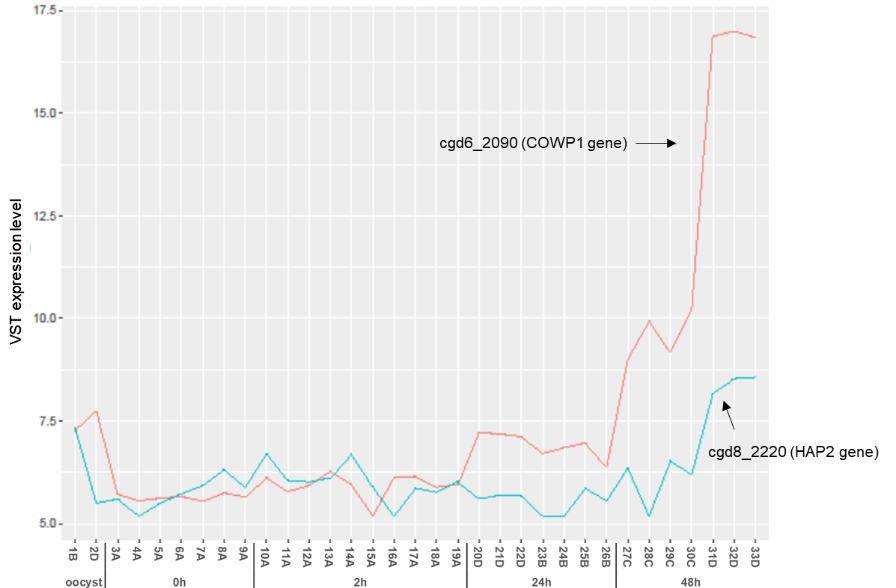**Supplementary Figure 1. Expression profile of sex-specific marker genes.** VST normalized expression levels of female marker COWP1 gene and male marker HAP2 gene in the 33 RNA-Seq samples are visualized. The sample ID is as indicated on the x-axis    **Supplementary Figure 2.**  **Expression profile of 48 h-specific genes.** VST normalized expression levels of 48 h-specific genes selected from the literature are visualized within the 33 RNA-Seq samples. The sample ID is as indicated on the x-axis.  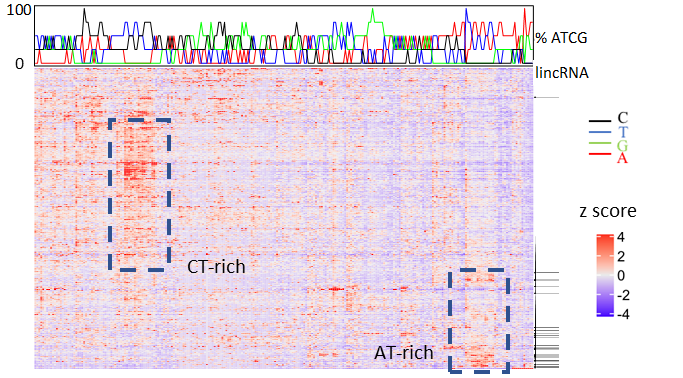  **Supplementary Figure 3. Relative 4-mer abundance profile of lncRNAs.** Each row represents a lncRNA candidate, and each column represents a 4-mer (4^4^=256 4-mers). The abundance of each 4-mer is indicated by color. Z score is calculated based on relative abundance with respect to mRNAs. Red suggests the 4-mer in lncRNAs is more abundant than in mRNAs. Blue indicates the opposite. The intergenic lncRNAs (lincRNAs) are indicated by the black line on the right , the remainder are antisense transcripts. The top panel shows the percent of ATCG for each 4-mer. From k-mer size 4 to 8, the clustering pattern remained but was less apparent. Thus, only results from 4-mer are shown here.     |
| --- |
| **Supplementary Figure 4. Significant motifs detected in the 100bp upstream region of lncRNAs.** The enriched motifs were returned from MEME v5.0.0 by searches of both strands of upstream lncRNA regions.  ****  **Supplementary Figure 5. Positional distribution of the five significant motifs in the 100 bp upstream region of lncRNAs.** The position of each motif was calculated as the start site of the motif relative to the transcription start site.  **** |

**Supplementary Figure 6. Visualization of select highly expressed lncRNAs.** RNA-Seq data from Batch D (HCT-8 samples) are shown. The coverage calculated from mapping data was normalized by CPM (counts per million reads mapped). The range shown here is 0-200 CPM. mRNA genes are named with the locus tag CPATCC. LncRNAs are named with Cp_lnc.
